# Cross-cultural adaptation, validity, and reliability of the Danish version of the neurophysiology of pain questionnaire

**DOI:** 10.1186/s41687-025-00899-w

**Published:** 2025-06-04

**Authors:** Nikolaj Agger, Thor Andre Brøndberg Stæhr, Michael Skovdal Rathleff, Lene Baad-Hansen, Shellie Boudreau, David Høyrup Christiansen

**Affiliations:** 1 Centre for Research in Health and Nursing, Regional Hospital Central Jutland, Viborg, Denmark; 2 Department of Occupational Medicine, University Research Clinic, Regional Hospital West Jutland, Herning, Denmark; 3 Elective Surgery Centre, Silkeborg Regional Hospital, Silkeborg, Denmark; 4 Center for General Practice at Aalborg University, Aalborg, Denmark; 5 Department of Health Science and Technology, Aalborg University, Aalborg, Denmark; 6 Center for Neuroplasticity and Pain, Center for Sensory Motor Interaction, Department of Health Science and Technology Faculty of Medicine, Aalborg University, Aalborg, Denmark; 7 Department of Dentistry and Oral Health, Aarhus University, Aarhus, Denmark; 8 Department of Clinical Medicine, Aarhus University, Aarhus, Denmark

**Keywords:** Pain education, Healthcare students, Neurophysiology of pain questionnaire, Validity, Reliability, Structural validity, Test-retest reliability, Translation, Cross-cultural adaptation.

## Abstract

**Background:**

Despite advances in medicine and technology, pain remains a significant global burden. Improving pain education for undergraduate healthcare students is considered an important step toward enhancing pain management. The Neurophysiology of Pain Questionnaire (NPQ) is commonly used to assess pain knowledge in healthcare students, but its validity and reliability in this population remain uncertain. This study aimed to translate and cross-culturally adapt the NPQ for Danish-speaking healthcare students and evaluate its measurement properties in Danish physiotherapy, medicine, and odontology students.

**Methods:**

The study was conducted in two phases: (1) translation and cross-cultural adaptation of the NPQ following international guidelines, and (2) a cross-sectional study to evaluate its validity and reliability in a sample of 224 Danish undergraduate healthcare students. Structural validity was assessed using exploratory factor analysis. Internal consistency was evaluated using Cronbach’s alpha, while test-retest reliability was determined using the intraclass correlation coefficient (ICC). Measurement error was analyzed using the standard error of measurement (SEM) and minimal detectable change (MDC).

**Results:**

Factor analysis revealed 11 factors, each with eigenvalues below 1, suggesting poor structural validity. Factor loadings were below the recommended threshold of 0.50, indicating weak item clustering. Internal consistency was low (Cronbach’s alpha = 0.37, 95% CI: 0.27 to 0.47), and test-retest reliability was poor (ICC = 0.39, 95% CI: 0.03 to 0.66). Measurement error analysis showed an SEM of 1.94 (95% CI: 1.54 to 2.63) and an MDC of 5.38 (95% CI: 4.27 to 7.28). No floor or ceiling effects were observed.

**Conclusions:**

The Danish version of the NPQ demonstrated poor structural validity, internal consistency, and reliability in undergraduate healthcare students. These findings raise concerns about its suitability for assessing pain neurophysiology knowledge in this population. Alternative tools or modifications to the NPQ may be necessary to improve its measurement properties.

## Introduction

Despite recent decades of medical and technological advancements, pain continues to be a significant societal burden worldwide [1]. The negative personal and societal impact underlines the need for improved pain care across the world [2].

Improving pain education for undergraduate healthcare students across disciplines is considered one potential step toward addressing the global burden of pain [3]. Healthcare professionals’ perspectives and beliefs regarding pain influence their clinical decision-making [4–6]. Consequently, there is a need for rigorous and reliable assessment of pain education programs for future healthcare professionals to ensure that their clinical practice aligns with the latest knowledge in pain management. Several measurement tools exist for evaluating pain knowledge in healthcare professionals [7], including the 19-item Neurophysiology of Pain Questionnaire (NPQ) [8, 9]. The tool has been used to assess healthcare students’ knowledge about pain in cross-sectional studies and intervention studies evaluating pain education programs [10–12]. Despite its common use to assess healthcare students’ knowledge about pain, the validity and reliability of the original NPQ have primarily been evaluated in patient populations suffering from pain conditions [9, 13–15]. To our knowledge, only two studies have evaluated the validity and reliability of NPQ with students as their target population: one conducted in Finland and one in Israel [16, 17], both reporting low internal consistency and only moderate test-retest reliability in student populations.

As the validity and reliability likely depend on population and contextual characteristics, evaluation should be conducted within the setting in which the questionnaire will be used [18]. Given its prior use in the Danish context and the availability of translated versions in other languages, the NPQ was considered a relevant instrument for adaptation and validation in a Danish educational setting. Thus, the aims of the present study were to (1) translate and cross-culturally adapt the NPQ for use in populations of Danish-speaking healthcare students and (2) evaluate the validity and reliability of the NPQ in Danish students of physiotherapy, medicine, and odontology.

## Methods

The present study was conducted in two steps. First, a translation and cross-cultural adaptation of the original English NPQ into a Danish version was completed. Secondly, the measurement properties of the NPQ were evaluated in a sample of undergraduate healthcare students (physiotherapy, medicine, and odontology).

### Part 1: Translation and cross-cultural adaptation

The original version of the NPQ was chosen, as it is the version used in educational settings in Denmark. It is also commonly used in other cross-cultural adaptations, allowing for comparison across languages. The NPQ was translated and cross-culturally adapted into Danish following international guidelines, following the ISPOR Principles of Good Practice for the Translation and Cultural Adaptation Process for Patient-Reported Outcomes Measures [19]. Permission to translate the NPQ was obtained from Mark Catley [9].

The questionnaire was translated from English to Danish by a professional English language translator specializing in medical translation and by an experienced pain researcher from the project group (LBH), both Danish native speakers. Subsequently, the project group and the professional translator reviewed both translations and compared them against the original English version at a consensus meeting to ensure conceptual and semantic equivalence.

The questionnaire was then back-translated from Danish to English by a professional native English-speaking translator without prior knowledge of the original version. The back-translation was compared to the original version by the professional translator and by two project group members (TS, SB). Afterwards, all project group members reviewed and approved the translation report.

Finally, cognitive debriefing interviews to assess comprehensibility were performed with 1st year (*n* = 3) and 3rd year (*n* = 4) physiotherapy students recruited from VIA University College Holstebro, Denmark. Individual interviews were conducted with the students, affording them the opportunity to express their opinions on how they understood each question. The outcomes of the interviews were compiled and condensed into a document. The cognitive debriefing results were reviewed in the project group, allowing adjustments to be made prior to finalizing the questionnaire.

### Part 2: Design and study population

The study was a cross-sectional questionnaire study encompassing Danish physiotherapy students from VIA University College Holstebro and medicine and odontology students from Aarhus University, Denmark. Students were required to understand Danish well enough to self-complete the questionnaire. Participants were informed about the project and gave informed consent by submitting their answers. Demographics (age, education, semester, and gender) and questionnaire data were collected between November 2019 and February 2020 using the Clinical Trial Unit at Aarhus University (https://redcap.au.dk/).

### Participants and data collection

Data collection was conducted by visiting lectures across the educational programs. All students present at the lectures completed the questionnaire. In total, 224 students completed the questionnaire online at the end of a lecture: 129 were 3rd year Medicine students, 48 were 4th-year odontology students, and 47 were physiotherapy students (*n* = 32 2nd-year and *n* = 15 3rd-year students). Only physiotherapy students were invited to complete the NPQ a second time for test-retest analysis. Of the 47 physiotherapy students who completed the initial round, 29 were selected to complete the questionnaire again after 7 to 10 days. The test and retest were administered under the same conditions, with students completing the questionnaire online at the end of scheduled lectures, using the same platform and instructions. This subset was selected due to practical constraints, such as time limitations and participant availability, while ensuring a sufficient sample size for test-retest reliability assessment.

### The neurophysiology of pain questionnaire

The NPQ is a 19-item questionnaire developed by Lorimer Moseley to assess knowledge about pain and the underlying neurophysiology in health care professionals and patients. Two versions of the NPQ exist: 1) the original NPQ [8] and 2) the modified NPQ [9], where some questions were changed based on a Rasch Analysis of the psychometric properties. The modified version also has a 12-item and 13-item version (sometimes called revised NPQ in the literature) [9]. The present study used the original version. The questionnaire has three response options (true, false, or undecided). Correct responses are scored 1, and incorrect responses (including the undecided option) are scored 0. Thus, scores can range from 0 to 19, with higher scores indicating greater knowledge about the neurophysiology of pain. As recommended by Catley et al., the participants were prompted to use the ‘undecided’ option instead of guessing [9].

### Statistical analysis

Stata, IC version 17.0 (College Station, Texas, USA) was used for all analyses. P-values of < 0.05 were considered statistically significant.

### Descriptive statistics

Descriptive statistics were calculated for variables age, gender, semester and NPQ score and compared between the three samples of healthcare students (physiotherapy, medicine, and odontology). Possible floor and ceiling effects were analyzed and were considered present if more than 15% of respondents scored the lowest or highest possible score.

### Structural validity and internal consistency

After confirming that the assumptions for exploratory factor analysis were met, the dimensionality of the questionnaire was assessed through an exploratory factor analysis of the total sample of healthcare students. Factors with eigenvalues greater than 1 were deemed significant, suggesting that the factor is significant in explaining the observed correlations among variables in the dataset. Factor loadings exceeding 0.50 were regarded as indicating strong associations between variables and factors [20]. Internal consistency was assessed by calculating Cronbach’s alpha for the total sample of healthcare students, with scores ranging from 0.7 to 0.9 indicating acceptable internal consistency [20, 21].

### Measurement error and test-retest reliability

Systematic measurement error between NPQ scores at baseline and retest was analyzed through Bland-Altman plot and paired t-tests for the physiotherapy students. Standard error of measurement (SEM) and minimal detectable change (MDC = 1.96 x √2 × SEM) were calculated to estimate random error. Test-retest reliability was evaluated by computing the intraclass correlation coefficient (ICC) through a two-way mixed-effects model, aiming to examine the consistency of individual scores. A value exceeding 0.75 was considered acceptable for group testing [21, 22]. Cohen’s Kappa with quadratic weights was used for single-item and sum-score agreement.

## Results

### Part 1: translation and cross-cultural adaptation

The original English and final version of the Danish NPQ can be found in Tables 1 and 2—the translation aimed to retain the original meaning of the items.

**Table 1:** Original version of the neurophysiology of pain questionnaire

Questions		True	False	Undecided
1.	Receptors on nerves work by opening ion channels (gates) in the wall of the nerve.	X		
2.	When part of your body is injured, special pain receptors convey the pain message to your brain.		X	
3.	Pain only occurs when you are injured.		X	
4.	The timing and intensity of pain matches the timing and number of signals in nociceptors (danger receptors).		X	
5.	Nerves have to connect a body part to your brain in order for that part to be in pain.		X	
6.	In chronic pain, the central nervous system becomes more sensitive to nociception (danger messages).	X		
7.	The body tells the brain when it is in pain.		X	
8.	The brain sends messages down your spinal cord that can increase the nociception (danger message) going up your spinal cord.	X		
9.	The brain decides when you will experience pain.	X		
10.	Nerves adapt by increasing their resting level of excitement.	X		
11.	Chronic pain means that an injury hasn’t healed properly.		X	
12.	Nerves can adapt by making more ion channels (gates).	X		
13.	Worse injuries always result in worse pain.		X	
14.	Nerves adapt by making ion channels (gates) stay open longer.	X		
15.	Second-order nociceptor (messenger nerve) post-synaptic membrane potential is dependent on descending modulation.	X		
16.	When you are injured, the environment that you are in will not have an effect on the amount of pain you experience.		X	
17.	It is possible to have pain and not know about it.		X	
18.	When you are injured, chemicals in your tissue can make nerves more sensitive.	X		
19.	In chronic pain, chemicals associated with stress can directly activate nociception pathways (danger messenger nerves).	X		

**Table 2:** Danish version of the neurophysiology of pain questionnaire

Spørgsmål		Sandt	Falsk	Ved ikke
1.	Receptorer på nerver fungerer ved at åbne ionkanaler (porte) i nervens cellevæg.	X		
2.	Når en del af din krop er skadet, bliver der sendt smertesignaler til din hjerne af særlige smertereceptorer.		X	
3.	Smerte opstår kun, når du er skadet.		X	
4.	Timingen og intensiteten af smerte svarer til timingen og antallet af signaler i nociceptorerne.		X	
5.	En kropsdel skal være forbundet til din hjerne med nerver, for at den pågældende kropsdel kan føle smerte.		X	
6.	Ved kronisk smerte bliver centralnervesystemet mere følsomt over for nociception.	X		
7.	Kroppen fortæller hjernen, når den har smerter.		X	
8.	Hjernen sender signaler ned gennem rygmarven, der kan øge den nociception, som er på vej op gennem rygmarven.	X		
9.	Hjernen afgør, hvornår du oplever smerte.	X		
10.	Nerver tilpasser sig ved at øge deres spændingsniveau i hvile.	X		
11.	Kronisk smerte betyder, at en skade ikke er helet ordentligt.		X	
12.	Nerver kan tilpasse sig ved at danne flere ionkanaler (porte).	X		
13.	Alvorligere skader medfører altid værre smerte.		X	
14.	Nerver tilpasser sig ved at lade ionkanaler (porte) stå åbne i længere tid.	X		
15.	Andenordens nociceptorers (modtagerneuroners) postsynaptiske membranpotentiale er afhængigt af descenderende modulation.	X		
16.	Det miljø, du befinder dig i, vil ikke påvirke graden af den smerte, du oplever, når du er skadet.		X	
17.	Det er muligt at have smerter uden at vide det.		X	
18.	Når du er skadet, kan signalstoffer i dit væv gøre nerver mere sensitive.	X		
19.	Ved kronisk smerte kan signalstoffer, der er forbundet med stress, aktivere de nociceptive baner direkte.	X		

A few minor disagreements arose during the translation process and were resolved through consensus. To improve linguistic clarity and cultural appropriateness, some items were rephrased and technical terms adapted. For instance, the English word “gates” was replaced with the Danish equivalent “porte” (e.g., Q1, Q12, Q14). Colloquial terms such as “faresignalreceptorer” were also removed (e.g., Q4, Q6, Q8, Q19) to maintain professional terminology.

The physiotherapy students participating in the cognitive debriefing found the NPQ acceptable and easy to complete (completion time < 6 min), and considered it a valid measure of their pain knowledge. Based on their feedback, Q16 was adjusted for clarity, as several students misinterpreted the phrasing of “will not have an effect.” The cognitive debriefing also confirmed the decision—supported by project group discussions—to remove parenthetical explanations intended for patients (e.g., “danger messenger nerves”), which were not suitable for an academic Danish context.

### Part 2: Descriptive statistics

The healthcare students’ characteristics and scores of the 224 healthcare students who completed the first round of the NPQ are presented in Table 3.

**Table 3:** Participant characteristics

	All	Physiotherapy	Medicine	Odontology	Test-retest subjects (physiotherapy)
n	224	47	129	48	29
Age, years (sd)	23.5 (2.8)	25.8 (4.1)	22.6 (1.8)	24.0 (1.8)	26.0 (4.9)
Gender, n (%)					
Female	165 (73.7)	24 (51.1)	98 (76.0)	43 (89.6)	17 (58.6)
Male	59 (26.3)	23 (49.9)	31 (24.9)	5 (10.4)	12 (41.4)
Year, n					
2nd year	32	32			17
3rd year	144	15	129		12
4th year	48			48	
NPQ, score (sd)	11 (2.26)	10.34 (2.40)	11.25 (2.10)	10.98 (2.45)	10.38 (2.53)
NPQ, round 2 score (sd)					10.80 (2.44)

Abbreviations: SD: standard deviation; NPQ: neurophysiology of pain questionnaire

The healthcare students had a mean age of 23.5 years (95% CI: 23.2; 23.9), with the medicine students being significantly younger than the physiotherapy and odontology students, while 74% of the participants were female. Slight differences in NPQ scores were observed between the three groups of students, with only the difference between physiotherapy and medical students being statistically significant. The percentage of correct answers on single items ranged from 4% in Q2 to 96% in Q3. No floor or ceiling effects were observed (scores ranging from 4 to 18).

### Structural validity and internal consistency

**Fig. 1:**
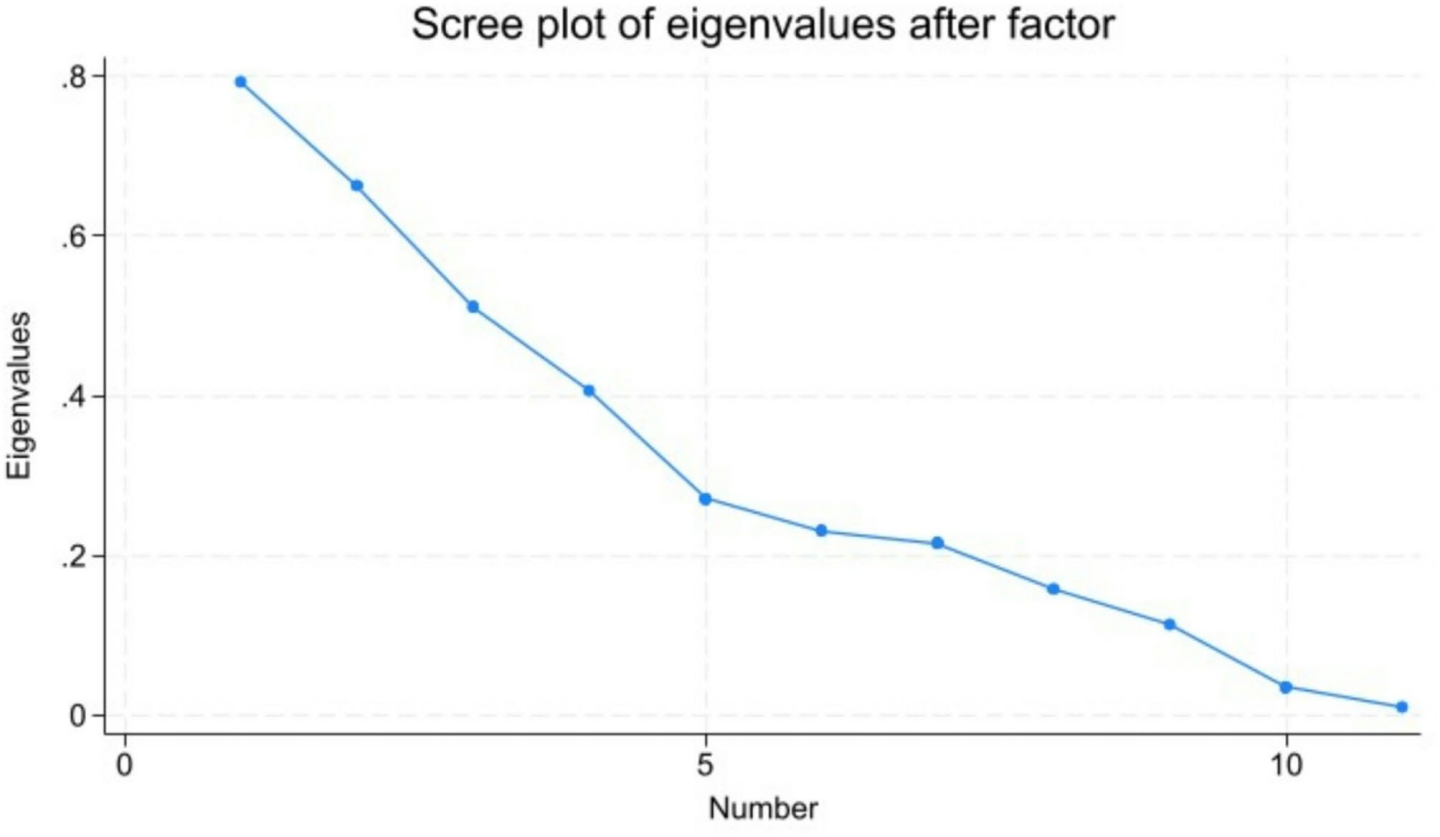
Scree plot of eigenvalues

For the total sample of students, the factor analysis did not generate any significant factors with an eigenvalue above the recommended 1 (see Fig. 1).

In total, 11 factors were generated with eigenvalues ranging from 0.01 to 0.79 and factor loadings of all items below the recommended 0.50 (see Table 4).

**Table 4:** Rotated factor loadings of factor analysis

Variable	Factor1	Factor2	Factor3	Factor4	Factor5	Factor6	Factor7	Factor8	Factor9	Factor10	Factor11
Q15	0.4184	-0.0037	-0.0262	0.0140	0.0285	0.0753	-0.0214	0.0699	0.1457	-0.0097	-0.0062
Q1	0.4029	0.0062	0.0920	-0.0727	-0.1160	-0.0316	-0.0606	0.0501	0.0073	0.0683	0.0279
Q8	0.3839	-0.0549	0.0211	0.0755	0.1028	-0.0126	0.1984	0.0225	-0.0865	-0.0386	-0.0195
Q19	-0.0729	0.4621	-0.0266	0.0346	-0.0675	0.0474	-0.0734	0.0076	0.0952	0.0271	0.0124
Q14	0.0937	0.3861	0.0357	-0.0220	0.1326	-0.0059	0.0448	0.1386	-0.0298	-0.0221	-0.0122
Q16	0.0984	-0.2440	0.0975	-0.0416	0.0067	0.0753	0.1085	0.0500	0.0463	-0.0884	0.1088
Q13	0.0446	-0.0080	0.4013	-0.0615	0.0854	0.0732	0.0994	0.1062	-0.0334	-0.0406	0.0280
Q5	0.0218	-0.0173	0.3919	0.1281	-0.0143	0.0399	-0.0854	-0.0591	0.1114	0.0520	-0.0255
Q3	-0.0067	0.0125	0.0592	0.3400	-0.0048	0.0639	0.0555	0.0198	0.0527	-0.0528	-0.0129
Q7	-0.0481	-0.0729	-0.0072	-0.3292	0.0029	0.1697	0.1329	0.0501	-0.0504	-0.0311	-0.0087
Q11	-0.0032	0.0056	0.0580	-0.0249	0.3841	0.0000	-0.0649	0.0515	0.1051	-0.0101	-0.0043
Q4	-0.0878	-0.0913	0.1244	0.1365	0.2326	0.1939	0.1445	0.0060	0.0918	-0.0040	0.0276
Q9	0.0273	0.0569	0.1023	-0.0252	0.0054	0.4129	0.0300	-0.0171	0.0060	-0.0008	0.0016
Q17	0.0897	0.0062	-0.1028	-0.1461	0.1873	0.2076	0.0960	-0.0422	0.0003	0.0930	-0.0058
Q2	0.0279	-0.0776	0.0104	-0.0431	-0.0474	0.0620	0.3829	0.0236	0.0111	0.0115	0.0050
Q12	0.1120	0.0817	0.0624	-0.0482	0.0706	-0.0098	0.0668	0.3672	0.0876	0.0187	-0.0004
Q18	0.2151	0.2074	-0.0278	0.1091	-0.0483	-0.0464	-0.1383	0.2597	-0.0699	-0.0902	0.0126
Q6	0.0792	0.1199	0.0763	0.0879	0.1615	0.0044	0.0077	0.0611	0.3582	0.0257	0.0034
Q10	0.0641	0.1560	0.0440	-0.0636	-0.0202	0.0274	0.0377	-0.0177	0.1530	0.2167	-0.0073

The Cronbach alpha coefficient, reflecting internal consistency, was 0.37 (95% CI: 0.27 to 0.47).

### Measurement error and test-retest reliability

The reliability statistics were calculated with the scores from 29 of the 47 physiotherapy students who completed the test-retest (see Table 5).

**Table 5:** Measurement error and reliability of the NPQ test-retest (*n* = 29)

**Measurement error**		
Mean difference (95% CI)	-0.41	(-1.46 to 0.63)
SEM (95% CI)	1.94	(1.54 to 2.63)
MDC (95% CI)	5.38	(4.27 to 7.28)
**Reliability**		
ICC (95% CI)	0.39	(0.03 to 0.66)
Single item agreement, Kappa, median (range)	0.39	(-0.13 to 0.76)
Sum-score agreement, Kappa (95% CI)	0.23	(-0.03 to 0.45)

Abbreviations: CI: confidence interval, SEM: standard error of measurement, MDC: minimal detectable change, ICC: interclass correlation coefficient

The analysis of measurement error indicated a mean difference of -0.41 [95% CI: -1.46 to 0.63]. The Standard Error of Measurement (SEM) was 1.94[95% CI: 1.54 to 2.63]. The Minimal Detectable Change (MDC) was 5.38[95% CI: 4.27 to 7.28].

Regarding reliability, the Intraclass Correlation Coefficient (ICC) was 0.39[95% CI: 0.03 to 0.66]. Single-item agreement, measured by Kappa, had a median value of 0.39, ranging from − 0.13 to 0.76, while sum-score agreement was 0.23[95% CI: -0.03 to 0.45].

## Discussion

The NPQ was translated and cross-culturally adapted to the Danish language, and its measurement properties were evaluated in a population of undergraduate physiotherapy, medicine, and odontology students. Our findings demonstrate that the Danish version of the NPQ has poor structural validity, internal consistency, and reliability levels for assessing knowledge about pain neurophysiology.

Despite extensive use for assessing healthcare students’ knowledge about pain, the present study is, to our knowledge, the first to evaluate the validity and reliability of the NPQ in a population of students while simultaneously evaluating the dimensionality and factor structure of the tool. Previously, two studies have investigated measurement properties of the original NPQ. They found Cronbach’s alphas of 0,77 [15] and 0,73 [17] and an ICC of 0,76 [15]. However, the authors did not evaluate the dimensionality and factor structure of the questionnaire. Understanding the dimensionality is crucial for ensuring the questionnaire’s reliability and validity in different settings, thus limiting the broader applicability and interpretability of the NPQ beyond the specific patient population studied.

The factor analysis revealed 11 factors, each with an eigenvalue below 1, indicating that none of these factors explained more variance than a single survey item. This suggests that the identified factors may not strongly represent the underlying constructs they were intended to measure, likely due to low inter-item correlations. We had anticipated that the NPQ would encompass at least three dimensions: nerve physiology, pain neurophysiology, and mechanisms/perception of pain. However, the weak factor structure suggests that the items did not cluster together as expected across these dimensions. Our hypothesis of multiple dimensions is consistent with findings from other studies [9, 10, 13, 14], where factor analyses of the modified NPQ indicated at least two factors. However, the presence of multiple weak factors introduces complexity, making it difficult to justify a single overall score as a valid measure of pain neurophysiology knowledge. Consequently, using the original NPQ to derive a total sum score may not provide a meaningful or reliable assessment of this construct.

The Cronbach’s Alpha value was 0.39, which is well below the recommended level [20] and lower than other studies reporting alpha values of 0.77 (original) [15], 0.44 (modified) [14], 0.52 (revised) [13], 0,73 [17] and 0.84 (modified) [9]. One possible explanation is the population differences (healthcare students vs. patients). The homogeneity of the student population compared to the heterogeneity of patient populations may contribute to a smaller deviation of data, potentially explaining the inherent differences. Consideration should also be given to the precision of the translations. However, the risk of inaccuracies in this process is considered as low, as the translation process was conducted by a project group with expertise in the field and adhered to international guidelines.

The test-retest reliability (ICC = 0.39) was poor and lower than in previous studies reporting ICCs of 0.76 (original) [15], 0.97 (modified) [9], 0.64 (modified) [14] and 0.48 (revised) [14]. The higher ICCs of the first two might be explained by the use of shorter time intervals of 24 h and 2 to 5 days between test and retest, increasing the likelihood of people remembering their previous answers. This would be more unlikely in the present study given the time interval of 7 to 10 days. In this study, there was no clear indication of a learning effect, as the mean difference between test and retest scores was small and not statistically significant (–0.41, 95% CI: − 1.46 to 0.63). Furthermore, participants received no feedback on their initial responses and were not encouraged to review or discuss the questionnaire between administrations. However, a learning effect cannot entirely be ruled out. Additionally, variations in study populations and data could contribute to the observed differences, given that ICC is responsive to different patterns of data deviation.

The large sample of undergraduate healthcare students with different educational backgrounds is likely representative of healthcare students of different knowledge levels in general. As recommended by the COSMIN guidelines [23], we intended to include at least 50 physiotherapy students in the test-retest, but this was not possible for practical reasons. A larger sample size would have led to narrower confidence intervals, potentially impacting the reliability statistics. However, we have confidence in the results of the exploratory factor analysis and calculation of internal consistency as the total sample of students (> 200) was well above the recommended sample size standards [23]. Descriptive statistics for the subgroup of physiotherapy students included in the test-retest analysis were not collected separately, which limits detailed subgroup characterization.

As the questionnaire is a test of knowledge, there is a natural risk of participants guessing, impacting the results of the analyses. To prevent this, participants were prompted to use the ‘undecided’ option instead of guessing, as recommended by Catley et al. and others [9, 14]. The undecided option was used by more than 28% on six questions (Q8, Q10, Q12, Q14, Q15, and Q19), mainly related to ‘nerve physiology’.

### Implications

The implications of this study raises concerns about the continued use of NPQ in Danish undergraduate students. The identified limitations underscore the need for caution, suggesting a reconsideration of alternative tools or potential adaptations. Given the observed deficiencies, a meticulous revision may be warranted to enhance its structural validity, internal consistency, and reliability, aligning it more closely with Danish healthcare students.

## Conclusion

The Danish version of the NPQ demonstrated poor measurement properties for assessing pain neurophysiology knowledge in undergraduate healthcare students. Therefore, using the questionnaire for testing this population cannot be recommended.

## Data Availability

In accordance with Danish regulations, access to the study dataset from which the reported findings in this paper are derived, is restricted and cannot be granted to other researchers.

## Abbreviations

NPQ: Neurophysiology of pain questionnaire
CI: Confidence interval
ICC: Intraclass correlation coefficient
SD: Standard deviation
MDC: Minimal detectable change
